# JOINT TRAJECTORIES OF SOCIAL ISOLATION AND LONELINESS AND THEIR EFFECTS ON ACCELERATED BIOLOGICAL AGING

**DOI:** 10.1093/geroni/igac059.715

**Published:** 2022-12-20

**Authors:** Xiang Qi, Daniel Belsky, Yang Yang, Ted Ng, Bei Wu

**Affiliations:** New York University, New York City, New York, United States; Mailman School of Public Health, Columbia University, New York City, New York, United States; University of North Carolina at Chapel Hill, Chapel Hill, North Carolina, United States; Arizona State University, Phoenix, Arizona, United States; New York University, New York, New York, United States

## Abstract

Social isolation and loneliness (SI/L) are associated with a variety of physical and mental health conditions. Biological aging is a proposed mechanism through which psychosocial factors drive health disparities. However, it is not known how SI/L separately and jointly affect biological aging. Using longitudinal data (N=1,965 adults, aged 50+) in Health and Retirement Study (2006 to 2016), we tested the joint trajectories of SI/L and their differential effects on biological aging as quantified by five DNA Methylation clocks (Horvath, Hannum, PhenoAge, GrimAge, and DunedinPoAm). A group-based mixture modeling approach was used to fit the joint trajectories of SI/L. Biological aging was measured using residuals from regressing each clock on chronological age. Linear regressions were used to test associations of joint trajectories of SI/L with biological aging. Four SI/L trajectories were identified, including Rarely isolated and rarely lonely (47.8%), Moderately isolated and rarely lonely (15.5%), Rarely isolated and severely lonely (20.2%), and Persistently isolated and lonely (16.5%). Of all joint trajectory groups, the Rarely isolated and rarely lonely group had the youngest biological age as estimated by the Hannum, PhenoAge, and GrimAge clocks. Compared with the Rarely isolated and severely lonely group, the Moderately isolated and rarely lonely group had higher age acceleration for PhenoAge (β=0.10; 95% CI=0.01, 0.19), and GrimAge (β=0.20; 95% CI=0.06,0.33), and DunedinPoAm (β=0.21; 95% CI=0.09,0.33). Different trajectories of SI/L convey differential risks to biological aging. Future research is needed to investigate the differences and similarities of SI/L trajectories and identify intervention targets for ameliorating aging.

